# Popcorn politics: Entertainment appraisals predict support for populist leaders

**DOI:** 10.1111/bjop.12791

**Published:** 2025-04-23

**Authors:** Jan‐Willem van Prooijen, Julia Kipperman, Yuxuan Li, Yifan Mo, Paul Nachtwey

**Affiliations:** ^1^ Department of Experimental and Applied Psychology Vrije Universiteit Amsterdam Amsterdam The Netherlands; ^2^ The Netherlands Institute for the Study of Crime and Law Enforcement Amsterdam The Netherlands; ^3^ Department of Criminal Law and Criminology Maastricht University Maastricht The Netherlands; ^4^ Department of Social Psychology Tilburg University Tilburg The Netherlands

**Keywords:** entertainment, political speech, popcorn politics, populism, support

## Abstract

Populism refers to a political style that describes society as a struggle between corrupt elites versus noble people and occurs across the political spectrum. What explains the appeal of populist leaders? In the present contribution, we tested the hypothesis that entertainment appraisals predict support more strongly for populist than non‐populist leaders. Four preregistered studies conducted among US participants supported this hypothesis, comparing appraisals of existing politicians between parties (Trump vs. Biden; Study 1) and within parties (Trump vs. Romney, Study 2a; Sanders vs. Biden, Study 2b). Furthermore, we experimentally exposed participants to a populist versus non‐populist speech of an unknown politician in a fictitious society (Study 3). Of importance, all studies also showed that the link between general populist attitudes and support was mediated by entertainment appraisals, but only for the relatively populist politicians. We conclude that to some extent, populism is a form of ‘popcorn politics’: Support for populist leaders depends on how entertaining people find them, more so than support for non‐populist leaders.

In recent years, populist movements have enjoyed remarkable electoral success in Europe (e.g., Fidesz in Hungary; Smer in Slovakia; PVV in the Netherlands), the US (Trump), Asia (e.g., Modi in India; Duterte in the Philippines) and Latin‐America (e.g., Bolsonaro in Brazil; Milei in Argentina). Populism is commonly defined as a political style that portrays society as a continuous struggle between the “elites” versus the “people”. This definition includes the elements of anti‐elitism (i.e., the belief that societal elites are corrupt and oppress the people) and people‐centrism (i.e., a focus on normal, hard‐working citizens who are perceived as homogenous, and uniformly represented by the populist leader) (Jagers & Walgrave, 2007; Mudde, 2004; Müller, 2016). What makes populist movements so appealing to many citizens? In the present research, we examine the possibility that to some extent, populism is a form of ‘popcorn politics’: Support for populist leaders depends on how entertaining people find them, more so than support for non‐populist leaders.

Populism has few unique ideological elements and has been referred to as a “thin‐layered” or “hollow” ideology that can be attached to various host ideologies (Mudde & Kaltwasser, 2017). As such, populist movements have emerged across the political spectrum, ranging from the far left (e.g., Occupy Wall Street; EU radical socialist parties) to the far right (e.g., MAGA; EU radical anti‐immigration parties). Due to their distinct host ideologies, important differences exist between left‐ and right‐wing populist movements. For instance, right‐wing populist movements tend to focus on reducing immigration and often are more authoritarian (Bernhard & Kriesi, 2019; Morelock, 2018). Left‐wing populist movements place a higher premium on the economic downsides of globalization, including a lower trust in multinationals and an increased concern for income equality (Akkerman et al., 2017; Obradovic et al., 2020; Rooduijn & Akkerman, 2017). What these different movements share, however, are anti‐elitism and people‐centrism as a key part of their rhetoric and underlying assumptions.

Prior research examining populist support has mostly focused on push factors: Negative sentiments that drive people towards populist movements. For instance, populist attitudes have been associated with symbolic and realistic threats (Abadi et al., 2024), insecurity (Kinvall, 2018; Van Prooijen, 2023), political cynicism and powerlessness (Papaioannou et al., 2023), anxiety (Jetten & Mols, 2021), disagreeableness (Bakker et al., 2021), anger (Magni, 2017; Rico et al., 2020), feelings of hate (Martinez et al., 2023), political discontent (Rooduijn et al., 2016), and protest (Schumacher & Rooduijn, 2013). Pull factors – positive sentiments that attract people to populist movements or leaders – have received less attention but are also important. For instance, populist movements offer epistemic clarity by proposing simple solutions for complex problems (Erisen et al., 2021; Van Prooijen et al., 2018; Van Prooijen & Krouwel, 2020), and relatedly, a reliance on common sense knowledge (Staerklé et al., 2022). Moreover, populist attitudes are associated with nostalgia, a complex emotional state that includes both negative and positive feelings (Mols & Jetten, 2014; Smeekes et al., 2018; Van Prooijen, Rosema, et al., 2022). Finally, populist rhetoric elicits not only negative but also positive emotions, including hope and pride (Wirz, 2018; see also Breeze, 2019). In the present contribution, we seek to add to this body of research by examining a pull factor for populist support that hitherto had not been recognized in empirical research: The extent to which people find a leader entertaining.

## Entertainment and populist support

Entertainment appraisals are defined as the extent to which people find a particular stimulus interesting, exciting, and attention‐grabbing. Such entertainment appraisals are associated more strongly with the intensity rather than the valence of emotions (Van Prooijen, Ligthart, et al., 2022). For instance, people may find a comedian entertaining by virtue of their ability to make them laugh; but likewise, people may find a scary movie entertaining by virtue of its ability to frighten them. Experiencing intense emotions decreases psychological distance (Van Boven et al., 2010) and can increase attraction to strangers (Dutton & Aron, 1974). As such, entertainment appraisals are likely to play a role in how people evaluate politicians.

In general, populist politicians often possess a range of features that could entertain large groups of citizens. Their anti‐elitism implies that populist politicians not only oppose mainstream politicians but also often openly question their morality and competence. This yields unpredictability and excitement by eliciting conflict, hence stirring up the established political order. Moreover, populist leaders frequently espouse a bleak worldview that makes assumptions about powerful and evil forces trying to harm society, often in the form of conspiracy theories (Erisen et al., 2021; Papaioannou et al., 2023; Van Prooijen, Cohen Rodrigues, et al., 2022). Research indicates that people find such conspiracy theories more entertaining than non‐conspiratorial narratives: Much like a detective novel, conspiracy theories imply mystery, suspected danger, and unexpected or unusual plot lines. Such entertainment appraisals are associated with people's willingness to believe these conspiracy theories (Van Prooijen, Ligthart, et al., 2022).

We propose that these entertaining qualities are functional for populist politicians in gaining support. Political speeches in general are likely to consist of various components designed to persuade voters, such as broad ideological statements, specific policy proposals, and the personal style of the politician (e.g., charisma; emotional appeals). Such personal style matters in political persuasion as political attitudes and political polarization are closely coupled with emotions (Webster & Albertson, 2022). One may wonder, however, if personal style has the same impact on people's support for populist versus non‐populist leaders. As populism itself has little unique ideological elements beyond the host ideology it is attached to (Mudde & Kaltwasser, 2017) and often offers relatively simplistic solutions for complex problems (Erisen et al., 2021), the persuasiveness of populist rhetoric may depend more strongly on heuristic cues than on substance. Personal style therefore is likely to matter more for populist than for non‐populist politicians.

This notion is consistent with previous work on the emotional basis of populist attitudes. From the supply side, populist rhetoric is more emotionally charged than non‐populist political rhetoric, and emphasizes both positive and negative emotions (Breeze, 2019). Correspondingly, from the demand side, populist speeches elicit stronger positive and negative emotions among perceivers (Wirz, 2018). Populist rhetoric thus puts a premium on intensified emotional experiences as a strategy to win the support of potential voters. Moreover, political communication via social media appears to benefit particularly populist parties. Although this is likely due to multiple reasons, one plausible factor is that social media (through user attention and algorithmic amplification) favours short, sensationalist, and emotional messages (Gerbaudo, 2018). Altogether, these arguments suggest that people's support for a populist politician might depend particularly on how entertaining they find the person. While any politician may benefit from being entertaining to some extent, our line of reasoning suggests that entertainment appraisals predict support more strongly for a populist than a non‐populist politician.

## The current research

The current research sought to test our line of reasoning with four studies. Studies 1, 2a, and 2b focused on existing politicians that are often seen as relatively populist versus relatively non‐populist and mainstream. Study 3 expanded on this by experimentally exposing participants to a populist or non‐populist speech by an unknown politician in a fictitious society.

Specifically, Study 1 was a between‐subjects study comparing US citizens who had voted for Trump versus Biden in the 2020 presidential elections, while empirically controlling for political ideology, education level, age, and gender. Studies 2a and 2b were within‐subjects designs comparing participants' ratings of politicians within the same party. Specifically, Study 2a consisted of Republican participants who evaluated Trump and Romney, while Study 2b consisted of Democratic participants who evaluated Sanders and Biden. Of course, as these are existing and well‐known politicians, base‐rate levels of entertainment are likely to differ (e.g., for a variety of reasons, one might expect that participants consider Trump more entertaining than Biden). Such a main effect is not our point, however. Our key hypothesis is that for the populist politician, expressed support will be associated more strongly with how entertaining participants find the person, as compared with the non‐populist politician.

Of course, only relying on existing politicians runs the risk of observing effects that may be due to idiosyncrasies of these specific individuals. We therefore extended the current research project in three ways. First, in Study 3 participants were exposed to an AI‐generated populist versus non‐populist speech taking place in a fictitious society. In Study 3 participants thus evaluated unknown politicians relying on a single speech, and we predicted that entertainment appraisals would predict support for the politician more strongly if participants were exposed to a populist than a non‐populist speech. Second, all studies included a validated measure of general populist attitudes (Akkerman et al., 2014). This enabled us to explore whether the effects observed here indeed are due to the populist features of the politicians under investigation. We specifically tested whether the link between populist attitudes and support would be mediated by entertainment appraisals, but only for the relatively populist politician. And third, in Study 3 we included measures of emotional intensity and emotional valence to acquire empirical evidence for our assumption that intense emotional experiences explain the link between entertainment appraisals and populist support.

## Open practices statement

All the studies reported here were preregistered, and we did not conduct other, undisclosed studies testing this hypothesis. The preregistrations, data, analysis scripts, questionnaires, and supplementary materials can be found on OSF.^1^ The data were analysed in *R* version 4.4.1. The studies have formal ethical approval as part of a cluster application by the first author.

## STUDY 1

In Study 1, US participants who voted for Trump in the 2020 elections rated how entertaining they found Trump, and to what extent they supported him; participants who voted for Biden rated these same variables for Biden. We predicted that entertainment appraisals would predict support for a politician, but also that this association would be stronger for Trump than for Biden.

### Method

#### Participants

We recruited a sample of 509 US participants (238 men, 262 women, 3 other; *M*_age_ = 40.54, *SD* = 13.62) through Prolific. We obtained 253 participants who indicated they had voted for Biden and 242 participants who indicated they had voted for Trump during the 2020 presidential elections, using Prolific's preselection tools. As preregistered, the remaining 14 participants (who indicated to not have voted, or to have voted for another candidate) were excluded from further analyses. The study lasted approximately 5 min and participants were paid 0.75 UKP (about 0.90 USD) for participation. Sensitivity analysis in G‐Power (Faul et al., 2009) indicates that a sample of 495 participants provides 80% power to detect a small effect size (*f* ^2^ = .016) for the predicted interaction.^2^

#### Procedure and materials

The study was conducted in January 2023. After providing their informed consent, participants first indicated who they had voted for in the 2020 US presidential election. Answers on the main variables of interest were recorded only for participants who indicated having voted for either Donald Trump or Joe Biden. Participants received the following questions only for the politician they had voted for.

Participants were asked to complete a measure of entertainment appraisals (cf. Van Prooijen, Ligthart, et al., 2022) by rating either Donald Trump or Joe Biden on the following 9 dimensions (1 = *not at all*, 5 = *very much*): interesting, entertaining, engaging, boring (recoded), dull (recoded), captivating, exciting, attention‐grabbing, and bland (recoded). Responses on these items were averaged into a reliable scale of entertainment appraisals (*α* = .96).

Participants subsequently indicated their support for Donald Trump or Joe Biden by answering four questions^3^ (1 = *not at all*, 5 = *very much*; example item: “Can you imagine supporting Donald Trump [Joe Biden] in a future election?”). Together these items formed a reliable scale of support for the politician (*α* = .87).

Participants then responded to the eight‐item populist attitudes scale (Akkerman et al., 2014; *M* = 3.91; *SD* = 0.66; *α* = .79). Example items were “The politicians in US congress need to follow the will of the people”, and “Elected officials talk too much and take too little action” (1 = *not at all*, 5 = *very much*).

After this, participants provided their basic demographics. We assessed political orientation with two items (1 = *very left‐wing*, 5 = *very right‐wing*, and 1 = *very liberal*, 5 = *very conservative*; *M* = 2.91; *SD* = 1.39; *α* = .98). At the end of the study, participants were thanked, debriefed, and redirected to a URL for payment.

### Results

Prior to analyses, vote choice was effect‐coded (−1 Trump, 1 Biden). Unsurprisingly, entertainment appraisals and vote choice were not independent: Trump was considered far more entertaining (*M* = 4.15, *SD* = 0.72) than Biden (*M* = 2.58, *SD* = 0.81), *F*(1, 492) = 516.07, *p* < .001; *η* ^2^ = .51. To avoid multicollinearity problems, we therefore mean‐centered the entertainment appraisals measure separately within both voter groups, thus reflecting participants' entertainment appraisals relative to other voters for the same politician. The interaction term was the product between vote choice and the mean‐centered entertainment measure.

#### Support for the politician

To test the hypothesis, we conducted a hierarchical linear regression with support for the politician as the dependent variable. As preregistered, Step 1 of our hierarchical regression model contained four control variables (gender, age, education level, and political orientation), Step 2 the main effects of vote choice and entertainment appraisals, and Step 3 the interaction. The results of this regression analysis are in Table 1. Step 1 was significant (*R* ^2^ = 0.03), *F*(4, 480) = 4.34, *p* < .001. Support for the politician was associated with older age and right‐wing political orientation. Step 2 added significantly to this model (Δ*R* ^2^ = 0.39), *F*(2, 478) = 163.46, *p* < .001, which was due to a strong main effect of entertainment appraisals. In general, people support a politician more to the extent they find the person more entertaining. The effect of vote choice was not significant, indicating that Trump and Biden received similar levels of support in our sample. More importantly, the predicted interaction that was added to Step 3 was significant (Δ*R* ^2^ = 0.03), *F*(1, 477) = 24.23, *p* < .001.

**TABLE 1:** Hierarchical regression analysis: Support for a politician as a function of entertainment appraisals and vote choice – Study 1.

Predictor	*B*	*SE*	*t*	CI_95%_
*Step1*				
Age	0.01	.00	2.29*	0.00; 0.01
Gender	0.04	.08	0.50	−0.12; 0.21
Education level	0.05	.05	1.02	−0.05; 0.16
Political orientation	0.08	.03	2.42*	0.01; 0.14
*Step 2*				
Entertainment appraisals	0.76	.04	17.85***	0.68; 0.84
Vote choice	0.06	.06	0.99	−0.06; 0.17
*Step 3*				
Interaction	−0.21	.04	−4.92***	−0.29; −0.13

* *p* < .05.

*** *p* < .001.

**FIGURE 1:**
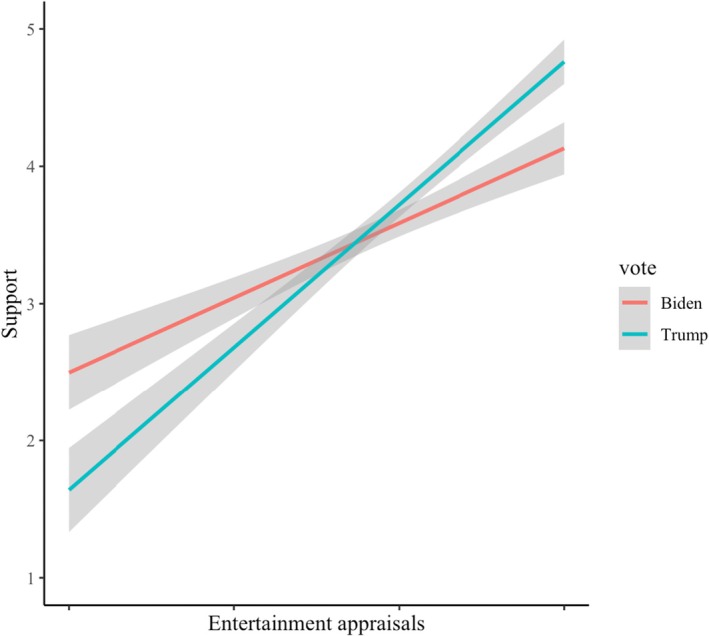
The interaction between entertainment appraisals and vote choice on support for a politician. Entertainment appraisals are mean centered separately for each voter group (Study 1).

The interaction is displayed graphically in Figure 1. We then proceeded to test the effect of entertainment appraisals on support, separately for Trump versus Biden voters. The slope of entertainment appraisals was steeper among Trump voters (*B* = 0.91, *SE* = .06, *p* < .001; CI_95%_[0.79; 1.04]) than among Biden voters (*B* = 0.58, *SE* = .06, *p* < .001; CI_95%_[0.47; 0.69]). Apparently, support for Trump depended more strongly on how entertaining voters found him than support for Biden. These findings supported our hypothesis.

#### Populist attitudes

We also examined the link between populist attitudes, entertainment appraisals, and support for the politician. Results indicated a populist attitudes × vote choice interaction on support, *B* = −0.19, *SE* = .06, *p* = .003; CI_95%_[−0.32; −0.05]. The association between populist attitudes and support was significant for Trump voters (*B* = 0.23, *SE* = .09, *p* = .008; CI_95%_[0.06; 0.41]) but not for Biden voters (*B* = −0.14, *SE* = .08, *p* = .107; CI_95%_[−0.30; 0.03]).

Our line of reasoning suggests that the link between populist attitudes and support would be mediated by entertainment appraisals, but only among Trump voters. We therefore conducted a moderated mediation analysis using *Rs* “mediation” package (Tingley et al., 2014). Results indicated a populist attitudes × vote choice interaction on entertainment appraisals, *B* = −0.20, *SE* = .05, *p* < .001; CI_95%_[−0.31; −0.10]. Consistent with our line of reasoning, the indirect effect was significant among Trump voters, *B* = 0.31, *SE* = .09, *p* < .001; CI_95%_[0.14; 0.48], but not among Biden voters, *B* = −0.06, *SE* = .05, *p* = .194; CI_95%_[−0.15; 0.03]. Populist attitudes positively predicted how entertaining participants found Trump, *B* = 0.33, *SE* = .06, *p* < .001; CI_95%_[0.20; 0.45], and these entertainment appraisals predicted support for Trump, *B* = 0.94, *SE* = .07, *p* < .001; CI_95%_[0.81; 1.07]. For Biden, populist attitudes were unrelated to entertainment appraisals, *B* = −0.10, *SE* = .08, *p* < .201; CI_95%_[−0.26; 0.06].

### Discussion

Study 1 yielded preliminary support for our line of reasoning. Entertainment appraisals predicted support for each politician; however, the association was stronger for Trump than for Biden. Also, the link between general populist attitudes and support was mediated by entertainment appraisals, but only among Trump voters. A clear limitation of Study 1, however, was that Trump and Biden differ on many dimensions, most notably party affiliation. In Studies 2a and 2b, we therefore implemented a within‐subjects design where Republican participants (Study 2a) and Democratic participants (Study 2b) evaluated two politicians within their own party that differ in the extent to which their style may be described as populistic.

## STUDIES 2A AND 2B

Study 2a contrasted Donald Trump with Mitt Romney – the first being commonly seen as a key example of a right‐wing populist, the latter being a well‐known former Republican presidential candidate and often seen as a more ‘traditional’ (non‐populist, and not part of the MAGA movement) Republican. Study 2b contrasted Bernie Sanders with Joe Biden. Discourse analyses have revealed that Sanders' rhetoric often emphasizes the antagonism between corrupt elites (e.g., the “1 percent”; greedy Wall Street CEOs) versus the common people and is therefore likely to appeal to left‐wing populist voters (Staufer, 2021). As with other left‐ versus right‐wing populist movements, many obvious differences exist between Trump and Sanders (and both criticize different societal elites); however, the elements of anti‐elitism and people‐centrism clearly emerges in the rhetoric of both politicians (see also Tucker Jr., 2022). In both studies, we empirically checked whether Trump and Sanders indeed appealed more strongly to people with relatively high populist attitudes within their respective parties.

Our first hypothesis was that the association between entertainment appraisals and support would be stronger for the relatively populist politicians (Trump and Sanders) than for the relatively mainstream politicians (Romney and Biden). Our second hypothesis was that the link between general populist attitudes and support for specifically Trump and Sanders would be mediated by entertainment appraisals.

### Method

#### Participants

We recruited a separate Republican (Study 2a) and Democratic (Study 2b) sample using Prolific's preselection tools. The Republican sample consisted of 350 US participants. We dropped three participants who indicated not to identify with the Republican party, leaving 347 participants for the analyses (206 men, 141 women; *M*_age_ = 43.71, *SD* = 13.71). The Democratic sample (Study 2b) consisted of 349 US participants; one of them indicated not to identify with the Democratic party, leaving 348 participants (157 men, 181 women, 10 non‐binary, 1 prefer not to say; *M*_age_ = 37.8, *SD* = 13.1). The study lasted about 5 min and participants received 0.75 UKP (about 0.90 US $). We aimed at recruiting around 350 participants per sample since correlations tend to stabilize around sample sizes of 250 participants, and we wanted to be on the safe side (Schönbrodt & Perugini, 2013; for a more detailed justification of the sample size, see the preregistration).

#### Procedure and measures

The two studies were run in parallel in February 2023. Both studies had a within‐subjects design. In the Republican sample, participants were asked about their opinions towards Donald Trump and Mitt Romney, and in the Democratic sample, participants were asked about their opinions towards Bernie Sanders and Joe Biden. After providing their informed consent, we first checked party affiliation by asking participants which political party they identified with the most. We then measured entertainment appraisals and support for each politician using the same measures as in Study 1 (1 = *not at all*, 5 = *very much*), while randomizing the order of politicians (entertainment appraisals: *α*_Trump_ = .92, *α*_Romney_ = .94 *α*_Sanders_ = .94, *α*_Biden_ = .94; support: *α*_Trump_ = .93, *α*_Romney_ = .94, *α*_Sanders_ = .92, *α*_Biden_ = .90).

Participants then responded to the same populist attitudes scale as Study 1 (1 = *not at all*, 5 = *very much*; Akkerman et al., 2014; Republican sample: *M* = 3.83, *SD* = 0.70, *α* = .81; Democratic sample: *M* = 3.77, *SD* = 0.65, *α* = .78). We also asked participants to indicate dichotomously who they prefer for an important office (such as President of the United States). The Republican sample largely preferred Trump over Romney (272 Donald Trump, 75 Mitt Romney), and the Democratic sample largely preferred Sanders over Biden (237 Bernie Sanders, 112 Joe Biden).

At the end of the survey, participants provided basic demographics and indicated their political orientation on the same two questions as in Study 1 (Republican sample: *M* = 4.17, *SD* = 0.59 *α* = .91; Democratic sample: *M* = 1.65, *SD* = 0.64, *α* = .82). Participants were thanked, debriefed, and redirected to a URL for payment.

### Results

#### Differences between politicians

Republican participants who preferred Trump for an important office scored higher on populist attitudes (*M* = 3.93, *SD* = 0.66) than those who preferred Romney (*M* = 3.46, *SD* = 0.73), *t*(345) = 5.36, *p* < .001, *d* = .90. Likewise, Democratic participants who preferred Sanders for an important office scored higher on populist attitudes (*M* = 3.90, *SD* = 0.61) than those who preferred Biden (*M* = 3.50, *SD* = 0.65), *t*(347) = 5.52, *p* < .001, *d* = .63. These findings support our assumption that Trump (Republican sample) and Sanders (Democratic sample) would be more likely to represent people relatively high in populist attitudes within their respective parties.

Furthermore, Republican participants found Trump more entertaining (*M* = 4.18, *SD* = 0.78) than Romney (*M* = 2.19, *SD* = 0.94), *t*(347) = 27.15, *p* < .001, *d* = 1.46. Democratic participants found Sanders more entertaining (*M* = 3.88, *SD* = 0.84) than Biden (*M* = 2.66, *SD* = 0.89), *t*(349) = 18.47, *p* < .001, *d* = 0.99. Consistent with Study 1, we therefore mean‐centered the four measures of entertainment appraisals for the subsequent analyses.

#### Support for the politician

Given that both studies had a within‐subjects design with two measures of entertainment appraisals and support for a politician, we tested our main hypothesis with linear mixed modelling using the lme4 package in *R*. The four control variables (gender, age, education level, and political orientation), the main effects of entertainment appraisals and politician, and the entertainment × politician interaction were entered as fixed effects, clustered within participant, which was added as a level 2 random effect.

**FIGURE 2:**
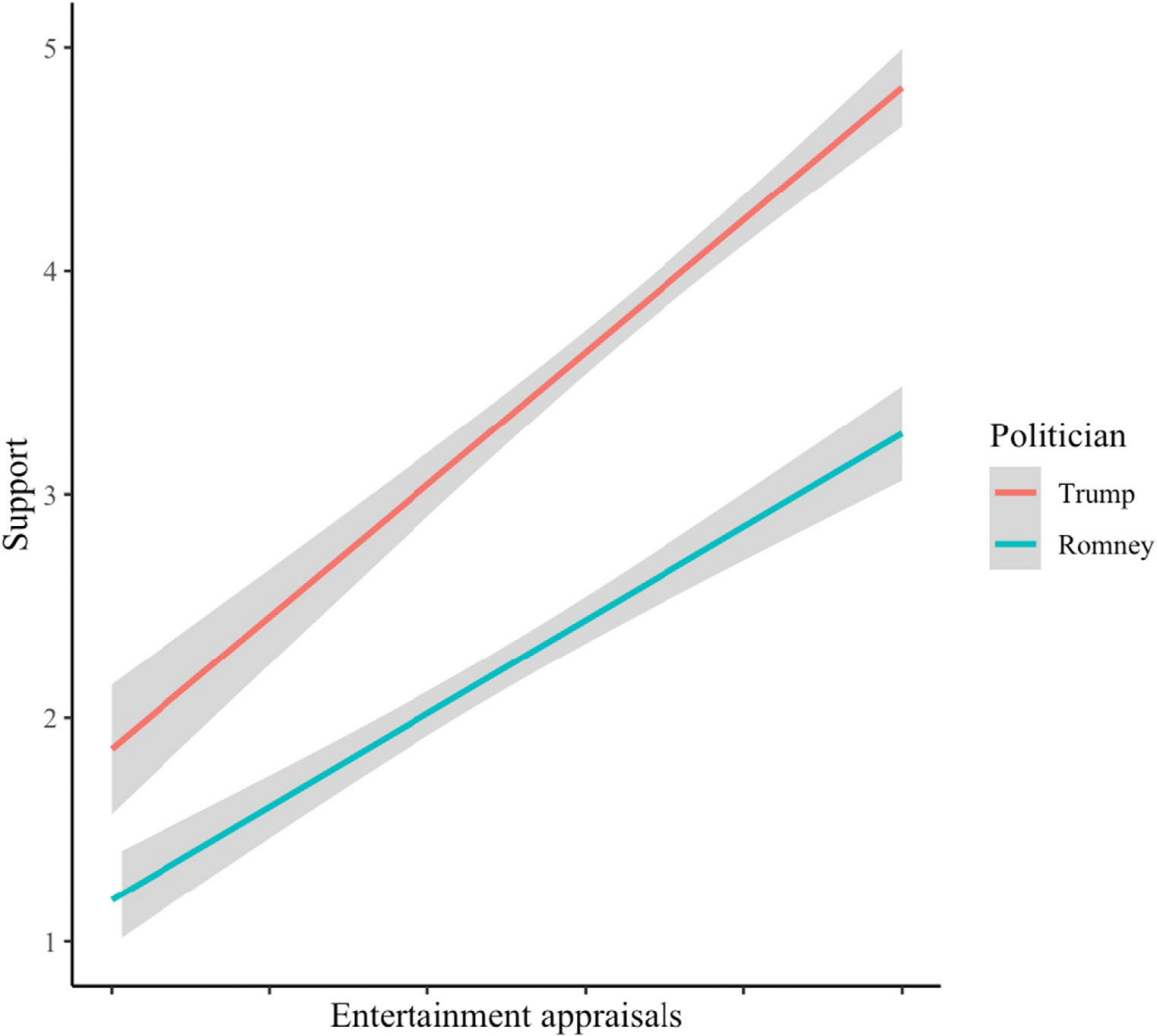
The relationship between entertainment appraisals of Trump and Romney on support for each politician. Entertainment appraisals are mean‐centered separately for each politician (Study 2a).

The results for both samples are displayed in Table 2. In the Republican sample (marginal *R* ^*2*^ = .57), none of the control variables was significant. The main effect of entertainment appraisals indicated that the more entertaining participants found a politician, the more strongly they supported him. The main effect of politician also was significant; participants supported Trump (*M* = 3.67, *SD* = 1.21) more than Romney (*M* = 2.39, *SD* = 1.11). More important was that the predicted entertainment × politician interaction was significant. The interaction is displayed in Figure 2. Consistent with our hypothesis, the association between entertainment appraisals and support was stronger for Trump (*B* = 1.01, *SE* = .06, *p* < .001; CI_95%_[0.89; 1.13]) than for Romney (*B* = 0.73, *SE* = .05, *p* < .001; CI_95%_[0.63; 0.83]).

**TABLE 2:** Linear mixed model results: Support for a politician as a function of entertainment appraisals, comparing Trump versus Romney (Republican sample, Study 2a) or Sanders versus Biden (Democratic Sample, Study 2b).

Predictor	Republican sample (2a)				Democratic sample (2b)			
	*B*	*SE*	*t*	CI_95%_	*B*	*SE*	*t*	CI_95%_
Gender	0.05	.07	0.71	−0.09; 0.19	0.06	.06	−0.93	−0.17; 0.06
Age	0.00	.00	−0.23	−0.01; 0.00	0.00	.00	0.49	0.00; 0.01
Education level	0.00	.04	−0.06	−0.08; 0.08	0.03	.04	0.84	−0.04; 0.10
Political orientation	0.08	.06	1.36	−0.03; 0.19	0.31	.05	−6.43***	−0.40; −0.21
Entertainment appraisals	0.90	.04	22.72***	0.82; 0.98	0.67	.04	19.25***	0.61; 0.74
Politician	0.64	.03	19.36***	0.58; 0.71	0.16	.03	5.48***	0.10; 0.22
Interaction	0.14	.04	3.57***	0.06; 0.22	0.06	.03	1.84*	0.00; 0.13

* *p* < .10.

*** *p* < .001.

**FIGURE 3:**
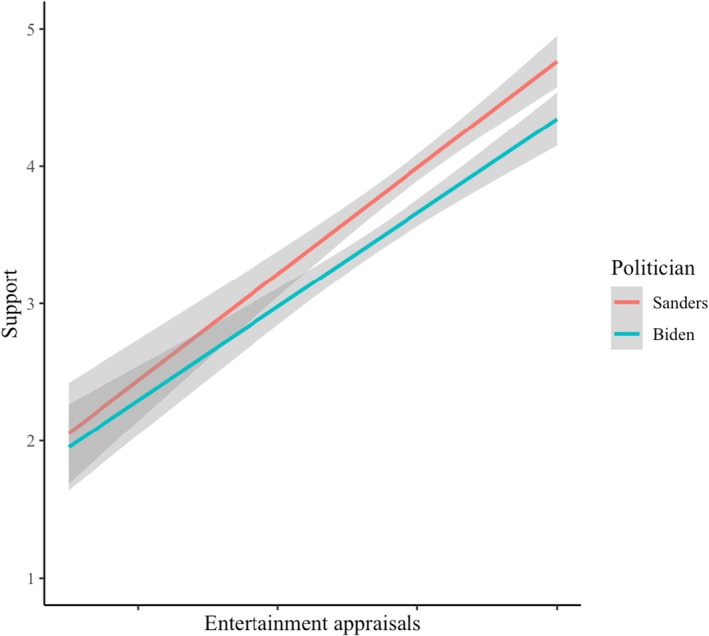
The relationship between entertainment appraisals of Sanders and Biden on support for each politician. Entertainment appraisals are mean‐centered separately for each politician (Study 2b).

In the Democratic sample (marginal *R* ^*2*^ = .42), stronger right‐wing orientation and entertainment appraisals predicted higher levels of support, and participants supported Sanders more strongly (*M* = 3.98, *SD* = 1.05) than Biden (*M* = 3.63, *SD* = 0.94). The predicted interaction was marginal (*p* = .067), although in the predicted direction, as the slope of entertainment appraisals appeared steeper for Sanders (*B* = 0.73, *SE* = .05, *p* < .001; CI_95%_[0.63; 0.83]) than for Biden (*B* = 0.53, *SE* = .05, *p* < .001; CI_95%_[0.44; 0.62]) (see Figure 3). Thus, our first hypothesis was supported in the Republican sample, but the findings were less conclusive in the Democratic sample.

#### Populist attitudes

In the Republican sample, we found a significant populist attitudes × politician interaction on support in a linear mixed model that included the control variables, *B* = 0.54, *SE* = 0.06, *p* < .001; CI_95%_[0.42; 0.65]. Populist attitudes predicted increased support for Trump (*B* = 0.49, *SE* = .09, *p* < .001; CI_95%_[0.32; 0.67]) and decreased support for Romney (*B* = −0.38, *SE* = .00, *p* < .001; CI_95%_[−0.55; −0.20]). Moreover, the interaction significantly predicted entertainment appraisals, *B* = 0.36, *SE* = 0.04, *p* < .001; CI_95%_[0.27; 0.45].

We then tested whether entertainment appraisals would mediate a link between populist attitudes and support for Trump, using the mediation package in *R* (Tingley et al., 2014). For Trump the indirect effect was significant, *B* = 0.30, *SE* = 0.06, *p* < .001; CI_95%_[0.18; 0.43]. Consistent with our second hypothesis, participants with stronger populist attitudes found Trump more entertaining, *B* = 0.31, *SE* = 0.06, *p* < .001; CI_95%_[0.19; 0.43], and these entertainment appraisals subsequently predicted increased support, *B* = 0.96, *SE* = 0.06, *p* < .001; CI_95%_[0.84; 1.09]. For Romney the indirect effect also was significant; however, the effect was negative, *B* = −0.24, *SE* = 0.06, *p* < .001; CI_95%_[−0.36; −0.12]. Participants with stronger populist attitudes found Romney *less* entertaining, *B* = −0.34, *SE* = 0.07, *p* < .001; CI_95%_[−0.48; −0.19], and such lower entertainment appraisals were associated with decreased support, *B* = 0.71, *SE* = 0.05, *p* < .001; CI_95%_[0.61; 0.81].

We followed the same analytic procedure in the Democratic sample. The populist attitudes × politician interaction on support was significant, *B* = 0.36, *SE* = 0.05, *p* < .001; CI_95%_[0.25; 0.47]. Populist attitudes were positively associated with support for Sanders (*B* = 0.35, *SE* = .08, *p* < .001; CI_95%_[0.19; 0.51]), and negatively associated with support for Biden (*B* = −0.22, *SE* = .07, *p* = .003; CI_95%_[−0.36; −0.07]). Also, the interaction was significant on entertainment appraisals, *B* = 0.18, *SE* = 0.05, *p* < .001; CI_95%_[0.09; 0.28].

The mediation analyses indicated a significant indirect effect for Sanders, *B* = 0.12, *SE* = 0.05, *p* = .017; CI_95%_[0.02; 0.22]. Consistent with our second hypothesis, populist attitudes were associated with finding Sanders more entertaining, *B* = 0.16, *SE* = 0.07, *p* = .021; CI_95%_[0.03; 0.30], which in turn predicted increased support for Sanders, *B* = 0.71, *SE* = 0.05, *p* < .001; CI_95%_[0.61; 0.81]. For Biden, the indirect effect was not significant, *B* = −0.05, *SE* = 0.04, *p* = .113; CI_95%_[−0.13; 0.01], as populist attitudes were not associated with entertainment appraisals, *B* = −0.10, *SE* = 0.07, *p* = .158; CI_95%_[−0.25; 0.04].

### Discussion

Study 2 examined our line of reasoning separately among Republicans (comparing Trump versus Romney) and Democrats (comparing Sanders versus Biden). The hypothesis that the link between general populist attitudes and support for specifically Trump (Study 2a) and Sanders (Study 2b) would be mediated by entertainment appraisals was supported in both samples. The hypothesis that the association between entertainment appraisals and support would be stronger for the relatively populist politician was supported in the Republican sample, but the crucial interaction effect was marginal in the Democratic sample. It is unclear how to interpret this latter difference in results between samples. One possibility is that the link between entertainment appraisals and populist support is generally more pronounced for right‐wing than left‐wing populist movements. An alternative possibility, however, is that these differences are related to idiosyncratic differences between these two well‐known politicians in the specific ways the public sees them as entertaining, the content of their messages, and the implications for support. As investigating differences between right‐wing versus left‐wing populist movements was not an explicit goal of the present project, we leave this issue for future research to resolve.

## STUDY 3

While the previous studies supported our line of reasoning, they are also limited by their focus on well‐known existing politicians that each have their own unique style. To prevent the current conclusions from being restricted to the specific politicians investigated here, in Study 3 we exposed participants to a populist versus a non‐populist speech of an unknown politician. The speeches were generated by AI and were situated in the context of a fictitious society. We tested the hypothesis that the extent to which participants found the speech entertaining would predict support for the politician, but particularly so in the populist speech condition. In a more exploratory fashion, Study 3 also included measures of emotional intensity and emotional valence. These measures aimed to explore a key assumption underlying our theoretical line of reasoning laid out in the introduction, namely that the entertaining qualities of populist rhetoric are rooted in their potential to elicit intense emotional experiences among people (see also Breeze, 2019; Wirz, 2018).

### Method

#### Participants

We recruited 598 US Prolific participants (339 men, 240 women, 11 non‐binary, 8 prefer not to say; *M*_age_ = 38.61, *SD* = 13.05). The study lasted about 6 min and participants were paid 0.90 UKP (about 1.25 US $). Participants were evenly distributed across the two conditions. We aimed at recruiting around 300 participants per condition, since our hypotheses predicted correlational associations between entertainment appraisals and support within conditions. As correlations stabilize at around 250 participants (Schönbrodt & Perugini, 2013), we slightly oversampled. This sample yields 80% power for detecting a small effect size for the predicted interaction (*f* ^2^ = .013).

#### Procedure and measures

The study was run in April 2023. After providing their informed consent, participants were asked to imagine living in a fictitious society called Zaloria, which was described as a diverse democracy that in recent years faced problems with job security. Participants were informed that a Zalorian politician gave a public speech addressing this issue during the current election campaign. We instructed participants to read the speech and afterward answer some questions about the speech and the politician. Participants were randomly assigned to read either a populist speech or a non‐populist speech (see Van Prooijen, Rosema, et al., 2022 for a similar procedure). We used OpenAI's ChatGPT to generate both speeches, although we critically evaluated (and slightly modified) the speeches ourselves to ensure they distinguished well between a populist versus a non‐populist narrative, according to the definition of populism.

In the populist speech, the politician blamed the elites of Zaloria for the problems of hard‐working citizens and presented themselves as a fighter for these citizens (example: “Our economy is rigged in favor of the wealthy and powerful, leaving the rest of us struggling to get by with low‐paying jobs, no benefits, and unstable employment.”). In the non‐populist speech, the politician focused less on blaming the elites and more on working together with everyone to create a prosperous economy (example: “In today's rapidly changing economy, it's more important than ever that we work together to create good‐paying jobs with strong benefits and protections.”; full text in the OSM). After reading the speeches participants were asked to briefly summarize them, and they responded to a comprehension check.

We measured entertainment appraisals of the speech with similar items as the previous studies, although we did tailor them to the specifics of this study. Specifically, as these were unknown politicians, we necessarily asked participants how entertaining they found the speech instead of the politician. The scale had excellent reliability (*α* = .96). Additionally, we asked participants to indicate on a slider how intense (0 = *not at all intense*; 100 = *very intense*) and how positive or negative (0 = *extremely negative*; 100 = *extremely positive*) their emotions were while reading the speech. We then assessed participants' support of the politician that gave the speech using five items (1 = *not at all*, 5 = *very muc*h; example item: “Would you like the politician to be the president of Zaloria?”, *α* = .92).

As a manipulation check, we asked participants to rate four statements on how much they applied to the politician that gave the speech. These statements referred to the anti‐elitist and people‐centric elements of populism (1 = *not at all*; 5 = *completely*; example item: “The politician blamed other Zalorian politicians”, *α* = .72).

Participants were informed that the subsequent questions would no longer concern Zaloria. We then administered the same populist attitudes scale as in previous studies (Akkerman et al., 2014; *α* = .76, *M* = 3.89, *SD* = 0.61), after which participants provided basic demographics and indicated their political orientation (*α* = .95, *M* = 2.43, *SD* = 1.12).

### Results

#### Manipulation check

A one‐way ANOVA on the manipulation check indicated that participants perceived the politician as more populistic in the populist speech condition (*M* = 3.94, *SD* = 0.64) than in the non‐populist speech condition (*M* = 2.40, *SD* = 0.57), *F*(1, 596) = 971.86, *p* < .001; *η* ^2^ = .62. This indicates that the manipulation was perceived as intended.

#### Support

We effect‐coded the conditions (1 populist speech, −1 non‐populist speech). As participants found the populist speech more entertaining (*M* = 3.36, *SD* = 0.64) than the non‐populist speech (*M* = 2.97, *SD* = 1.05), *F*(1, 601) = 23.16, *p* < .001; *η* ^2^ = .04, we mean‐centered the entertainment appraisals measure separately for each condition. The interaction was determined by calculating the product between the effect‐coded conditions and the mean‐centered entertainment measure. Given that participants were assigned randomly to conditions and the materials did not refer to existing politicians (and following our preregistered analysis protocol), we did not include control variables in the analyses; however, including the same control variables as the previous studies yielded similar results.

**FIGURE 4:**
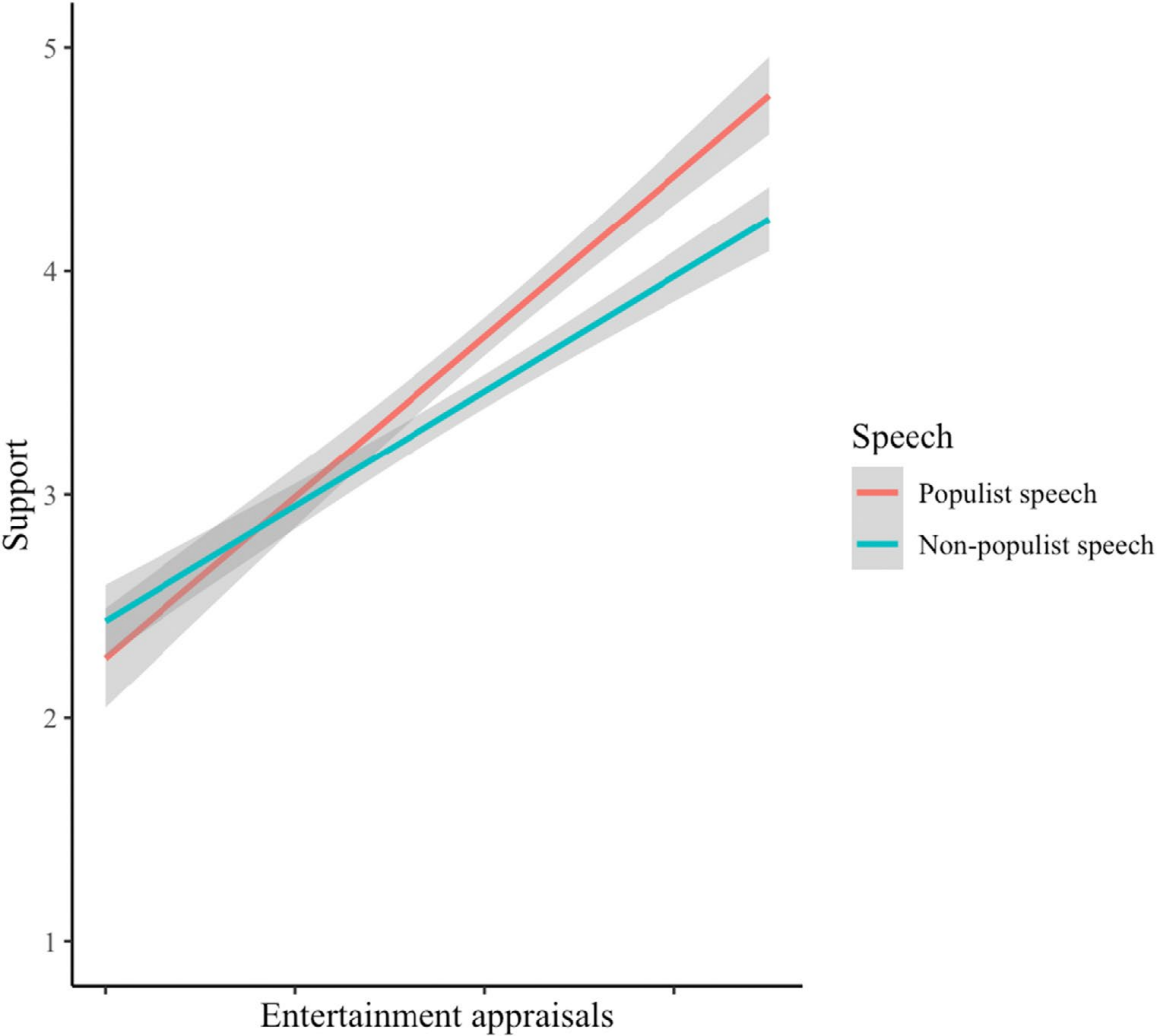
The relationship between entertainment appraisals of speech condition on support for the politician. Entertainment appraisals are mean‐centred separately for each condition (Study 3).

A hierarchical regression analysis showed that Step 1 (which included the two main effects) was significant (*R* ^2^ = 0.47), *F*(2, 596) = 269.35, *p* < .001. Entertainment appraisals were associated with increased support, *B* = 0.63, *SE* = 0.03, *p* < .001; CI_95%_[0.57; 0.68]. Moreover, people supported the politician more in the populist speech condition (*M* = 3.71, *SD* = 0.99) than in the non‐populist speech condition (*M* = 3.48, *SD* = 0.85), *B* = 0.11, *SE* = 0.03, *p* < .001; CI_95%_[0.06; 0.17]. Step 2 added significantly to the model (Δ*R* ^2^ = 0.01), *F*(1, 595) = 10.00, *p* = .002, yielding a significant interaction term, *B* = 0.09, *SE* = 0.03, *p* = .002; CI_95%_[0.03; 0.14]. As predicted, the slope of entertainment appraisals was steeper in the populist speech condition (*B* = 0.72, *SE* = 0.04, *p* < .001; CI_95%_[0.64; 0.80]) than in the non‐populist speech condition (*B* = 0.55, *SE* = 0.03, *p* < .001; CI_95%_[0.48; 0.62]). The interaction is displayed in Figure 4.

#### Populist attitudes

We first tested whether populist attitudes would predict support particularly in the populist speech condition. The populist attitudes × speech interaction was significant (*B* = 0.23, *SE* = 0.06, *p* < .001; CI_95%_[0.11; 0.35]). The link between populist attitudes and support was significant in the populist speech condition (*B* = 0.60, *SE* = 0.09, *p* < .001; CI_95%_[0.41; 0.78]) and nonsignificant in the non‐populist speech condition (*B* = 0.13, *SE* = 0.08, *p* = .082; CI_95%_[−0.02; 0.29]).

We then conducted a moderated mediation analysis. We found a significant populist attitudes × speech interaction on entertainment appraisals (*B* = 0.18, *SE* = 0.07, *p* = .006; CI_95%_[0.05; 0.31]). In the populist speech condition, the indirect effect was significant (*B* = 0.31, *SE* = 0.07, *p* < .001; CI_95%_[0.18; 0.45]). Populist attitudes predicted increased entertainment appraisals (*B* = 0.46, *SE* = 0.09, *p* < .001; CI_95%_[0.28; 0.64]), and these entertainment appraisals were significantly associated with support (*B* = 0.67, *SE* = 0.04, *p* < .001; CI_95%_[0.59; 0.76]). In the non‐populist speech condition, the indirect effect was not significant (*B* = 0.05, *SE* = 0.05, *p* = .319; CI_95%_[−0.05; 0.16]), as populist attitudes were unrelated to entertainment appraisals (*B* = 0.00, *SE* = 0.10, *p* = .324; CI_95%_[−0.09; 0.28])

#### Emotional intensity

We then explored the role of intense emotional experiences (independent from emotional valence) in these processes. Participants experienced more intense emotions when reading the populist speech (*M* = 53.11, *SD* = 26.89) than when reading the non‐populist speech (*M* = 41.52, *SD* = 26.97), *F*(1, 600) = 27.84, *p* < .001; *η* ^2^ = .04. The effect of the speech manipulation on emotional valence was not significant, *F*(1, 600) = 1.20, *p* = .274; *η* ^2^ = .00. Participants experienced moderately positive emotions while reading one of the speeches (overall *M* = 62.83, *SD* = 22.84).

We then mean‐centered the emotional intensity measure separately per condition and calculated the interaction term with the speech manipulation. After controlling for emotional valence and the main effects, this interaction significantly predicted support (*B* = 0.01, *SE* = 0.001, *p* < .001; CI_95%_[0.008; 0.013]). Emotional intensity had a steeper slope in the populist speech condition (*B* = 0.015, *SE* = 0.002, *p* < .001; CI_95%_[0.011; 0.019]) than in the non‐populist speech condition (*B* = 0.005, *SE* = 0.002, *p* < .001; CI_95%_[0.002; 0.008]). Apparently, support for the populist politician depended more strongly on the intensity of participants' emotions than support for the non‐populist politician.

Next, we examined whether the link between entertainment appraisals and support was mediated by emotional intensity, while controlling for emotional valence. This was the case in the populist speech condition (*B* = 0.12, *SE* = 0.05, *p* = .020; CI_95%_[0.02; 0.22]). Entertainment appraisals predicted emotional intensity (*B* = 19.64, *SE* = 1.24, *p* < .001; CI_95%_[17.20; 22.08]), and such intensity predicted support for the politician that delivered a populist speech (*B* = 0.006, *SE* = 0.002, *p* = .010; CI_95%_[0.001; 0.010]). In the non‐populist speech condition, the indirect effect was not significant (*B* = −0.001, *SE* = 0.03, *p* = .998; CI_95%_[−0.07; 0.06]). While entertainment appraisals were associated with emotional intensity (*B* = 17.19, *SE* = 1.41, *p* < .001; CI_95%_[14.41; 19.96]), such emotional intensity was not associated with support (*B* = 0.000, *SE* = 0.002, *p* = .978; CI_95%_[−0.004; 0.003]). In sum, consistent with prior research, entertainment appraisals were closely coupled with intense emotional experiences (Van Prooijen, Ligthart, et al., 2022). Such emotional intensity only predicted support for a politician who gave a populist (and not a non‐populist) speech, however.

### Discussion

Study 3 provided further support for our line of reasoning by experimentally manipulating exposure to a populist versus a non‐populist speech by an unknown politician. Also, Study 3 provided preliminary evidence for a role of emotional intensity in these processes, suggesting that the link between entertainment appraisals and support is attributable to the potential of populist rhetoric to elicit intense emotions among people.

## GENERAL DISCUSSION

The present research was designed to test whether being entertaining matters for the support that people give to politicians, and whether particularly populist politicians benefit from such entertainment appraisals. The results of four studies were consistent with this idea. In Study 1, entertainment appraisals were more strongly associated with voters' support for Trump than voters' support for Biden. In Study 2a, entertainment appraisals predicted support more strongly for Trump than for Romney among Republican participants; In Study 2b, entertainment appraisals predicted support (albeit marginally) more strongly for Sanders than for Biden among Democratic participants. In Study 3, entertainment appraisals predicted support more strongly when an unknown politician gave a populist than a non‐populist speech. In all studies, the link between general populist attitudes and support was mediated by entertainment appraisals, but only for the relatively populist politicians. These studies jointly suggest that, as compared with non‐populist politicians, populist politicians may electorally benefit more from being considered entertaining. Also, particularly people high in populist attitudes seem sensitive to the entertainment value of these politicians.

These findings make various novel contributions to the psychology of populism. First, whereas previous research and theorizing have overwhelmingly focused on push‐factors such as anxiety, insecurity, threat, hate, and related constructs (Abadi et al., 2024; Bakker et al., 2021; Jetten & Mols, 2021; Kinvall, 2018; Martinez et al., 2023; Rooduijn et al., 2016; Van Prooijen, 2023), the current findings underscore that pull‐factors also are important. These two notions are not in conflict and indeed might be closely related. For instance, one plausible possibility is that distressing societal circumstances (a push‐factor) make people more susceptible to relatively superficial traits of a leader that contribute to their entertainment value, such as their charisma, eloquence, or appeal to emotions. Future research could expand on the current findings by examining this possibility.

Second, the present findings fit in a broader line of research suggesting that a desire for entertainment is important to understand political beliefs, particularly radical ones. For example, conspiracy beliefs are associated with a dispositional aversion to boredom (Brotherton & Eser, 2015) and with the extent to which people find a particular conspiracy theory entertaining (Van Prooijen, Ligthart, et al., 2022). Moreover, the extent to which people are drawn to extreme political beliefs or groups involves closely related processes. Both dispositional and experimentally induced boredom predict more extreme political orientations at both the left and right (van Tilburg & Igou, 2016). Furthermore, sensation seeking is one possible motivation to join violent extremist groups (Nussio, 2020) and is associated with a stronger willingness to use violence in support of political goals (Schumpe et al., 2020). To some extent, radicalism is associated with a craving for excitement, and the present findings suggest that these notions may generalize to populist attitudes and support for populist leaders.

Finally, prior research on push‐factors mostly focuses on negative emotions to predict populist attitudes (e.g., Jetten & Mols, 2021; Magni, 2017; Rico et al., 2020; Rooduijn et al., 2016). The present findings suggest that independent of (negative) valence, the intensity of emotions is important in explaining populist support. This insight is consistent with earlier findings that populist rhetoric arouses both negative and positive emotions in people (Wirz, 2018). Relatedly, entertainment appraisals are associated with emotional intensity, and emotional intensity is a better predictor of conspiracy beliefs than emotional valence (Van Prooijen, Ligthart, et al., 2022). At the same time, we must acknowledge having examined emotional intensity as an exploratory variable in only one of the current studies (Study 3). While the results were promising, future research would need to more specifically focus on the role of emotional intensity in populist attitudes or support for populist leaders.

### Strengths and limitations

The current studies have several noteworthy methodological strengths. All studies were preregistered and well‐powered. Moreover, the studies yielded remarkably consistent results. Finally, while some studies had relatively high ecological validity by focusing on existing politicians, the last study had relatively high internal validity by experimentally manipulating exposure to a populist vs. non‐populist speech. Altogether, the studies presented here provide solid evidence that populist support is rooted more strongly in entertainment appraisals than non‐populist support.

We also need to acknowledge several limitations, however. First, Studies 1, 2a, and 2b exclusively examined appraisals of US politicians in a sample of US participants, and although Study 3 was situated in a fictitious context, this study also relied on a US participant sample. Populism is a global phenomenon, however, and may take different shapes and forms across the world. For instance, while Studies 2a and 2b focused on right‐wing and left‐wing populism in the US (i.e., Donald Trump and Bernie Sanders, respectively), some populist movements elsewhere are difficult to place on a left–right continuum and combine traditional left‐wing ideas with traditional right‐wing ideas (a case in point being Italy's Five Star Movement, that for instance argues for a universal basic income for all citizens, but also for strict immigration policies). Future research would need to examine to what extent the present conclusions generalize to populist movements elsewhere in the world.

Another limitation is that the present studies do not allow for causal conclusions. Although exposure to a populist speech was manipulated experimentally in Study 3, in all studies entertainment appraisals and support were measured variables. The analyses testing if entertainment appraisals would mediate the link between populist attitudes and populist support also are entirely correlational, and it is impossible to exclude another causal order than the one we assumed in our theoretical model. For example, people might find those leaders entertaining that they already supported. Future research may include longitudinal designs to test how the link between entertainment appraisals and populist support unfolds over time and experimental designs that manipulate how entertaining a particular speech is to establish its causal effects.

## CONCLUDING REMARKS

Throughout the world, populist movements have been on the rise. The leaders of these movements often are eccentric, attention‐grabbing individuals who regularly make controversial statements and stir up the political establishment by eliciting conflict. The present findings suggest that these entertaining features have an electoral function. Particularly for populist leaders, public support is associated with how entertaining people find them. One might speculate that the quality of governance therefore could vary more for populist than non‐populist leaders, as the populist style may successfully distract voters from the content of the proposed policies. To some extent, populism may be referred to as a form of popcorn politics.

## AUTHOR CONTRIBUTIONS

**Jan‐Willem van Prooijen:** Conceptualization; investigation; writing – original draft; methodology; visualization; writing – review and editing; supervision; formal analysis. **Julia Kipperman:** Conceptualization; investigation; methodology; writing – review and editing; formal analysis. **Yuxuan Li:** Conceptualization; investigation; methodology; writing – review and editing; formal analysis. **Yifan Mo:** Conceptualization; investigation; methodology; writing – review and editing; formal analysis. **Paul Nachtwey:** Conceptualization; investigation; methodology; writing – review and editing; formal analysis.

## Supporting information


**Data S1:** Supporting Information.

## Data Availability

All studies reported in this contribution were preregistered, and all data, analysis scripts, and materials are openly available on OSF (see also footnote 1 in the manuscript). Data, analysis scripts, materials, supplementary materials, and preregistrations Studies 1, 2a, and 2b: https://osf.io/bfjk2/. Preregistration Study 3: https://osf.io/7s2xw.

## Footnotes

^1^
Data, analysis scripts, materials, supplementary materials, and preregistrations Studies 1, 2a, and 2b: https://osf.io/bfjk2/. Preregistration Study 3: https://osf.io/7s2xw

^2^
Across studies, degrees of freedom differ slightly from the total sample due to attrition. To facilitate inclusion of gender as a control variable, a response “other” was coded as missing value. Study 1 also contained an exploratory measure of sensation seeking; details in the OSM.

^3^
The original preregistered scale contained six items. However, due to an unfortunate copy‐and‐paste error in the stimulus materials (accidentally associating Joe Biden with the Republican party) we had to drop two items.
