# Supplementary material for: Popcorn politics: Entertainment appraisals predict support for populist leaders
Source: Br J Psychol. 2025 Apr 23;116(4):770–88. doi: 10.1111/bjop.12791 (PMC12514324; doi:10.1111/bjop.12791)
Supplement: Supplementary file 1 — Data S1: Supporting Information. [file BJOP-116-770-s001.docx]

**Popcorn Politics:**

**Entertainment Appraisals Predict Support for Populist Leaders**

ONLINE SUPPLEMENTARY MATERIALS

**Materials Study 1**

**Vote choice:**

Who did you vote for in the 2020 election?

- Donald Trump (1)
- Joe Biden (2)
- Other (3)
- Did not vote (4)

**Entertainment appraisals – Trump / Biden**

To what extent do you think Donald Trump / Joe Biden is…  1(not at all) to 5 (very much)

Interesting; entertaining; engaging; boring; dull; captivating; exciting; attention-grabbing; bland

**Support for politician:**

Please rate the following questions about your support for Donald Trump / Joe Biden (1= not at all, 5= very much)

- Can you imagine supporting Donald Trump / Joe Biden in a future election?
- Do you mostly support policies proposed by Donald Trump / Joe Biden?
- Do you agree with the viewpoints of Donald Trump / Joe Biden?
- Do you want Donald Trump / Joe Biden to be the president of the United States?

(In Study 1 two additional items were dropped due to an unfortunate copy-and-paste error, accidentally associating Joe Biden with the Republican party: “Would you rather have someone else represent the Republican party than Donald Trump / Joe Biden?” and “If the Republican party nominates him, will you vote for Donald Trump / Joe Biden in 2024?”. In Studies 2a, 2b, and 3, we did not re-include these two items but measured support with the four-items scale)

**Populist attitudes (Akkerman et al., 2014):**

To what extent do you agree with the following statements? (1= not at all, 5= very much)

- The politicians in US congress need to follow the will of the people
- The people, and not politicians, should make our most important policy decisions
- The political differences between the elite and the people are larger than the differences between the people
- I would rather be represented by a citizen than by a specialized politician
- Elected officials talk too much and take too little action
- What people call “compromise” in politics is really just selling out on one’s principles.
- Politics is ultimately a struggle between good and evil
- Interest groups have too much influence over political decisions

**Sensation seeking:**

To what extent do you agree with the following statement? (1= not at all, 5= very much)

- I would like to explore strange places
- I get restless when I spend too much time at home
- I like to do frightening things
- I like wild parties
- I would like to take off on a trip with no pre-planned routes or timetables
- I prefer friends who are excitingly unpredictable
- I would like to try bungee jumping
- I would love to have new and exciting experiences, even if they are illegal

In politics people sometimes talk about “left” and “right”. Where would you place yourself on a scale from 1=very left-wing to 5=very right-wing?

- 1 = Very left-wing (1)
- 2 = Somewhat left-wing (2)
- 3 = Neutral (3)
- 4 = Somewhat right-wing (4)
- 5 = Very right-wing (5)

In politics people sometimes talk about “liberal” and “conservative”. Where would you place yourself on a scale from 1=very liberal to 5=very conservative?

- 1 = Very liberal (1)
- 2 = Somewhat liberal (2)
- 3 = Neutral (3)
- 4 = Somewhat conservative (4)
- 5 = Very conservative (5)

What is your age in years?

________________________________________________________________

Q9 Which gender do you identify with?

- Male (1)
- Female (2)
- Other (3)

Q10 Which is your highest education?

- no formal education (1)
- primary level education (2)
- secondary level education (3)
- College education (Bachelor’s degree) (4)
- College education (Graduate degree) (5)
- PhD (6)

**Materials – Study 2a and Study 2b**

party Which political party do you most identify with?

- the Republican Party (1)
- the Democratic Party (2)
- Others (3)

**Entertainment appraisals – Trump / Romney (Study 2a), Sanders / Biden (Study 2b)**

To what extent do you think Donald Trump / Mitt Romney / Bernie Sanders / Joe Biden is…  1(not at all) to 5 (very much)

Interesting; entertaining; engaging; boring; dull; captivating; exciting; attention-grabbing; bland

**Support for politician:**

Please rate the following questions about your support Donald Trump / Mitt Romney / Bernie Sanders / Joe Biden (1= not at all, 5= very much)

- Can you imagine supporting Donald Trump / Mitt Romney / Bernie Sanders / Joe Biden in a future election?
- Do you mostly support policies proposed by Donald Trump / Mitt Romney / Bernie Sanders / Joe Biden?
- Do you agree with the viewpoints of Donald Trump / Mitt Romney / Bernie Sanders / Joe Biden?
- Do you want Donald Trump / Mitt Romney / Bernie Sanders / Joe Biden to be the president of the United States?

**Populist attitudes**

This was measured with the same scale by Akkerman et al. (2014) as in Study 1.

DT_MR_forced If you could choose between these two candidates for an important office (such as president of the USA), who would you prefer?

- Donald Trump (1)
- Mitt Romney (2)

BS_JB_forced If you could choose between these two candidates for an important office (such as president of the USA), who would you prefer?

- Bernie Sanders (1)
- Joe Biden (2)

age What is your age in years?

________________________________________________________________

gender Which gender do you identify with?

- Male (1)
- Female (2)
- Non-binary / third gender (3)
- Prefer not to say (4)

edu Which is your highest education?

- no formal education (1)
- primary level education (2)
- secondary level education (3)
- College education (Bachelor’s degree) (4)
- College education (Master's degree) (5)
- Ph.D. Degree (6)

L/C In politics people sometimes talk about “liberal” and “conservative”. Where would you place yourself on a scale from 1=very liberal to 5=very conservative?

- 1 = Very liberal (1)
- 2 = Somewhat liberal (2)
- 3 = Neutral (3)
- 4 = Somewhat conservative (4)
- 5 = Very conservative (5)

L/R In politics people sometimes talk about “left” and “right”. Where would you place yourself on a scale from 1=very left-wing to 5=very right-wing?

- 1 = Very left-wing (1)
- 2 = Somewhat left-wing (2)
- 3 = Neutral (3)
- 4 = Somewhat right-wing (4)
- 5 = Very right-wing (5)

**Materials – Study 3**

**Manipulation:**

Please imagine you are living in a country called Zaloria. Zaloria is a diverse, multi-party democracy that holds elections every four years.  

In recent years, increasing numbers of people are experiencing job insecurity. During the current election campaigns, a Zalorian politician has addressed this issue in a recent public speech.

Please read the speech. Afterward, you will answer some questions about the politician who gave the speech.

***Populist speech condition***

The following paragraphs are a speech from a Zalorian politician, imagining you are the citizens of Zaloria.

"My fellow Zalorians,

I'm here to talk about a fundamental issue that's been ignored for too long: job security. Our economy is rigged in favor of the wealthy and powerful, leaving the rest of us struggling to get by with low-paying jobs, no benefits, and unstable employment. That's not fair, and it's not right.

The politicians in Zaloria have sold us out to the highest bidder, letting corporations ship our jobs overseas and exploit workers for their own gain. It's time we took back control and demanded that our government works for us, not just the wealthy elite.

I will fight tirelessly for the rights of working-class Zalorians. I'll fight for fair wages, affordable healthcare, and secure jobs that can support families. I'll stand up to the big corporations that have been robbing us blind for too long. And together, we'll build a better, more prosperous Zaloria."

sum_pop Please briefly summarize the speech you have just read (1-3 sentences).

________________________________________________________________

________________________________________________________________

________________________________________________________________

________________________________________________________________

________________________________________________________________

| 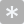 |
| --- |

forced_pop Does the politician promise to fight for the working class in the struggle with the wealthy elites?

- Yes (1)
- No (2)

***Non-populist speech***

The following paragraphs are a speech from a Zalorian politician, imagining you are the citizens of Zaloria.

"My fellow citizens,

We all know how important job security is to our families and our communities. In today's rapidly changing economy, it's more important than ever that we work together to create good-paying jobs with strong benefits and protections.

That's why I'm committed to supporting businesses that invest in their workers and communities. We need to ensure that workers have the skills and education they need to succeed in the jobs of the future. We also need to create an environment that fosters innovation and entrepreneurship, so that small businesses can thrive and create good jobs for their workers.

I believe that by working together, we can create an economy that works for everyone. That means providing a safety net for those who may experience job loss or insecurity, and promoting policies that support workers' rights and protections. We can build a future where all Zalorians have the opportunity to achieve success and prosperity."

sum_non-pop Please briefly summarize the speech you have just read (1-3 sentences).

________________________________________________________________

________________________________________________________________

________________________________________________________________

________________________________________________________________

________________________________________________________________

| 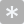 |
| --- |

forced_non_pop Does the politician promise to work together with everyone for prospering economy?

- Yes (1)
- No (2)

**Entertainment appraisals**

To what extent do you think the speech is…  1 (not at all) to 5 (very much)

Interesting; entertaining; engaging; boring; dull; captivating; exciting; attention-grabbing; bland

**Emotional intensity and emotional valence**

Please indicate how intense the emotions were that you felt while reading the speech (regardless of whether the emotions were positive or negative:
0 (not at all intense) - 100 (very intense).

|  | 0 | 10 | 20 | 30 | 40 | 50 | 60 | 70 | 80 | 90 | 100 |
| --- | --- | --- | --- | --- | --- | --- | --- | --- | --- | --- | --- |

| Your response () | 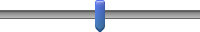 |
| --- | --- |

Please indicate how negative or positive the emotions were that you felt while reading the speech:
0 (very negative) - 100 (very positive).

|  | Extremely negative | Somewhat negative | Neither positive nor negative | Somewhat positive | Extremely positive |
| --- | --- | --- | --- | --- | --- |

|  | 0 | 10 | 20 | 30 | 40 | 50 | 60 | 70 | 80 | 90 | 100 |
| --- | --- | --- | --- | --- | --- | --- | --- | --- | --- | --- | --- |

| Your response () | 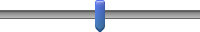 |
| --- | --- |

**Support**

Please rate the following questions about your support for the Zalorian politician who gave the speech, imagining you are the citizens of Zaloria. (1= not at all, 5= very much)

- Can you imagine supporting this politician?
- Would you support policies proposed by the politician?
- Would you like the politician to be the president of Zaloria?
- Would you rather have someone else be the president of Zaloria?
- How likely would you vote for this politician in a Zalorian election?

**Manipulation check**

To what extent do you think the following statements apply to the politician whose speech you read earlier? (1= not at all, 5= very much)

- The politician blamed other Zalorian politicians
- Referred to the common people of Zaloria
- Present himself as part of the Zalorian working class
- Present Zaloria as divided into two groups: the working class and the wealthy elites

**Populist attitudes**

Thank you for answering these questions. The following questions no longer concern Zaloria; instead they are concerned with your views about the real world.

Populist attitudes subsequently were measured with the same scale by Akkerman et al. (2014) as in Studies 1, 2a, and 2b.

**Demographics**

These were measured the same as Studies 2a and 2b.
